# Derivation of a novel clinical phenotype of sepsis in critically ill patients: a secondary analysis of a multicentre, prospective, observational study

**DOI:** 10.3389/fmed.2026.1910023

**Published:** 2026-09-16

**Authors:** YuTao Zha, LiangWen Cui, ZhuSheng Nie, Rui Huang, Cheng Zhang, Liang Zhou, Ming Fang, XiaoChun Peng, Jie Zhang, Heng Wang, Chao Yu, Nian Liu, Min Shao

**Affiliations:** 1 Department of Critical Care Medicine, The First Affiliated Hospital of Anhui Medical University, Hefei, Anhui, China; 2 Department of Biostatistics, Anhui Provincial Cancer Institute, The First Affiliated Hospital of Anhui Medical University, Hefei, Anhui, China; 3 Information Center, The First Affiliated Hospital of Anhui Medical University, Hefei, Anhui, China; 4 Center for Big Data and Population Health of IHM, Anhui Medical University, Hefei, Anhui, China

**Keywords:** clinical phenotype, derivation cohort, fluid resuscitation, machine learning, mortality, sepsis

## Abstract

**Background:**

Sepsis is a highly heterogeneous syndrome. Providing personalized treatment for the various clinical phenotypes of sepsis has been a major challenge for critical care physicians.

**Methods:**

This study was a secondary analysis of data derived from a multicenter, prospective, observational study of sepsis patients. Based on relevance to sepsis onset or outcome, 25 parameters were selected by the derived cohort to derive sepsis phenotypes. Clustered phenotypes were derived via k-means clustering and examined in a sensitivity analysis using divisive hierarchical clustering. Then, the relationship between fluid resuscitation therapy and survival outcomes was assessed for each clustered phenotype using generalized estimating equations.

**Results:**

Patients with sepsis were categorized into Cluster A, B and C. Kaplan–Meier analysis showed Cluster A patients had the highest 28-day mortality (*P* = 0.029 by Log-rank test). In Cluster A, septic patients who received fluid resuscitation had a association than those who did not, with an adjusted RD for 28-day mortality of 16.2% (95% CI, 1.4 to 31.0%, *P* = 0.032). Sensitivity analyses adjusted for APACHE II and infection site confirmed these findings, with an adjusted RD for 28-day mortality of 6.3% (95% CI, 0.3 to 12.3%, *P* = 0.040). Hierarchical clustering methods yielded consistent conclusions.

**Conclusion:**

The association between fluid resuscitation and clinical outcomes varied across sepsis phenotypes, suggesting potential heterogeneity in treatment response that requires further prospective validation.

**Trial registration:** The primary study was registered in the China Clinical Registry on February 1, 2022, with the registration number ChiCTR2200056179.

## Introduction

Recent research on sepsis has indicated that its definition is overly broad, encompassing a vast array of clinical and biological features, which complicates its diagnosis and treatment. Sepsis is a heterogeneous syndrome, and it is unrealistic to expect that a single drug will provide therapeutic benefit throughout the entire disease process (1). The high mortality rate associated with sepsis is not solely due to ineffective drug therapy but is more attributable to the lack of individualized treatment for each patient (2). The complex etiology of sepsis and the wide variation in patients' immune responses, underlying diseases, and disease progression necessitate precise treatment tailored to each patient's unique situation.

Artificial intelligence and machine learning techniques can be employed to quantify similarities among individuals within sepsis populations, distinguishing various phenotypes through clustering analysis. This analysis considers not only temperature, hemodynamics, or the type of organ dysfunction but also fluid status/reactivity, trajectory, and outcome (3). Different phenotypes may respond distinctively to therapeutic strategies, and precisely defining the clinical characteristics of sepsis heterogeneity could be the initial step in our understanding of potential treatments.

Most existing phenotypes were derived from retrospective datasets in high-income settings, and their applicability to prospective ICU populations in China remains uncertain. It is also unclear whether routinely available variables collected during the first 24 h of ICU admission can identify clinically meaningful phenotypes associated with differential responses to fluid resuscitation. This study conducted a multicenter, prospective, observational investigation into sepsis among critically ill patients in Anhui Province, China. The purpose was to assess sepsis phenotypes and explore the heterogeneity of treatment outcomes. It explores the identification of sepsis clinical phenotypes and its potential to be combined with artificial intelligence and machine learning techniques in Anhui Province, with the expectation of providing theoretical foundations and practical guidance for improving treatment outcomes and survival rates of sepsis patients in low- and middle-income countries.

## Methods

### Data source

This study was a secondary analysis based on data obtained from a multicenter, prospective, observational study. The primary study focused on the diagnosis, treatment, and prognosis of critically ill patients, which was registered in the China Clinical Registry on February 1, 2022, with the registration number ChiCTR2200056179. The complete methodology of the original study is described in the Supplementary Appendix.

### Eligibility criteria

Inclusion Criteria: Age ≥18 years old, Sepsis 3.0 diagnostic criteria (according to Sepsis-3.0, the diagnostic criteria for sepsis are suspected or confirmed infection and acute elevation of SOFA score ≥2 points), Patient or family members agreed to participate in this study. Exclusion criteria: Expected ICU admission less than 24 h, End-stage malignant tumor.

### Outcomes

The primary outcome was death at 28 days from ICU admission. Secondary outcomes were the need for mechanical ventilation use of vasopressors and length of ICU stay.

### Statistical analysis

#### Clinical variables of phenotype

We selected 25 variables, based on associations with sepsis pathogenesis or outcome, for inclusion in a conceptual model of sepsis pathophysiology and host tolerance, available in the course record at the hospital visit. These include demographic variables such as age, gender, and body mass index (BMI), vital signs such as heart rate, respiratory rate, systolic blood pressure, and temperature, and markers of inflammation such as white blood cell (WBC), C-Reactive Protein (CRP), and procalcitonin (PCT). Markers of organ dysfunction or injury, such as Alanine aminotransferase (ALT), Aspartate aminotransferase (AST), Total bilirubin (TBIL), Blood urea nitrogen (BUN), Creatinine (Cr), Cardiac troponin I (cTnI), Lactate (Lac) and Arterial oxygen tension/Fraction of inspiration O_2_ (PO_2_/FiO_2_), coagulation indices such as prothrombin time (PT), activated partial thromboplastin time (APTT), platelet (PLT) and Fibrinogen (FIB) as well as Serum sodium (Natrium, Na), Red blood cell (RBC) and Albumin (ALB). For each variable, the worst value recorded within 24 h of admission to the ICU was collected. The variables included in the clustering analysis were prespecified based on a clinically informed framework reflecting key domains of sepsis heterogeneity, including demographics, physiological parameters, inflammation, organ dysfunction, tissue perfusion, coagulation, and metabolic/nutritional status. Variables were selected based on their clinical relevance, routine availability in the early phase of ICU admission, and potential ability to capture distinct biological phenotypes of sepsis. No outcome-based variable selection was performed to avoid outcome leakage and to ensure that the derived clusters represented clinical phenotypes rather than prognostic models.

#### Clinical phenotype derivation

The study assessed the distribution and missingness of phenotypic variables. Nonnormal data were log-transformed and scaled. Patients without 28-day mortality information were excluded, and all parameters with ≥30% missing data were excluded, excluding highly missing [erythrocyte sedimentation rate and inflammatory factors (interleukin-1, interleukin-6, interleukin-8, interleukin-10, and tumor necrosis factor, among others)]. Missing data were estimated for each study cohort using the random forest method using the missForest package (4). Random Forest interpolation is a nonparametric algorithm that accommodates nonlinearities and interactions without specifying a specific parametric model (5). Missing values were imputed using the default settings of the missForest package without additional tuning. The maximum number of iterations was 10, with 100 trees per random forest and mtry = floor(sqrt(p)). The algorithm stopped when the imputation difference increased or after 10 iterations, retaining the previous imputation. Imputation performance was assessed using OOB error: NRMSE for continuous variables and PFC for categorical variables. Random forests use bootstrap aggregation of multiple regression trees to reduce the risk of overfitting and incorporate many tree estimates (4). The missing values for patient baseline data, laboratory indicators and study outcomes are in Supplementary Table S1.

The sepsis phenotype was derived using Euclidean distance-based K-means, a fundamental and widely used machine learning-based clustering method (6). The optimal number of clusters was then determined using a consensus clustering method (7), which quantitatively and intuitively assesses the number of unsupervised classes in a dataset by inducing sampling variability in subsampling. In consensus clustering, Elbow method cumulative distribution function (CDF) and cluster-consensus map are used to assess the separation of consensus matrix heat map. In this study, K-means clustering method was visualized using Principal components factor analysis (PCA) (8) to assess clustering and plot the fragmentation map to reduce the dimensionality and visualize the high dimensional dataset. Divisive hierarchical clustering (DHC) was used as an alternative to K-means to derive phenotypes to confirm cluster consistency. The number of clusters was determined using Elbow method and Gap statistic methods (9).

#### Evaluating the association of fluid resuscitation on a cohort of sepsis clinical phenotypes

All eligible patients were treated with fluid resuscitation and other septic shock-related regimens according to the 2016 and 2018 International Save Sepsis Campaign Guidelines for the Management of Severe Sepsis and Septic Shock (10), which included the following: (1) measurement of lactate levels and review if initial lactate was ≥2 mmol/L, (2) before antibiotics were administered retention of blood cultures, (3) administration of broad-spectrum antibiotics, (4) rapid administration of 30 mL/kg of crystalloid, and (5) application of vasoactive medications to maintain a mean arterial pressure > 65 mmHg if hypotension persists during or after aggressive fluid resuscitation, (6) others including infusion of crystalloid, appropriate antibiotic therapy, infection source control, and organ-supportive therapy as directed by the attending physician.

To asses the association between fluid resuscitation and each clinical outcome, generalized estimating equations were used to adjust for hospital-level variance. These associations were analyzed after adjusting for potential confounders such as age, gender, Charlson comorbidity index (CCI) and Sequential organ failure assessment (SOFA) score. Cluster-level associations of fluid resuscitation were examined by adding an interaction term “fluid resuscitation use -×- cluster” to the model to test for differential associations of fluid resuscitation across clusters. Cluster-specific risk differences were reported for descriptive purposes, whereas the interaction *P* value was used as the formal test of association modification. For sensitivity analyses, we used generalized estimating equations with the Acute Physiology and Chronic Health Evaluation II (APACHE II) and site of infection as adjusting variables instead of the SOFA score. To further evaluate the robustness of the association between fluid resuscitation and clinical outcomes, a propensity score-based overlap weighting analysis was performed as a sensitivity analysis. Propensity scores were estimated using logistic regression models incorporating age, sex, Charlson Comorbidity Index, and SOFA score. Overlap weights were subsequently applied to generate a weighted population with improved balance between patients receiving and not receiving fluid resuscitation. Covariate balance before and after weighting was assessed using standardized mean differences and Love plots. These adjustment and weighting approaches address measured covariates only and cannot eliminate residual confounding from unrecorded treatment-selection factors.

Continuous variables were expressed using median (interquartile spacing) or mean ± standard deviation, and categorical data were expressed as numbers (percentages). Continuous variables were compared using t-tests or rank-sum tests. The chi-square test was used for the comparison of categorical data. Cumulative mortality rates were represented by probability plots, and differences were tested by the log-rank test. *p* < 0.05 was considered statistically significant. All statistical analyses in this study were performed using R Studio 4.4.1 (RStudio, Boston) software. The missForest, ConsensusClusterPlus, cluster, geepack, survival, WeightIt, survey, and cobalt packages were used for data imputation, clustering, regression, survival, and weighting analyses. A fixed random seed was set using set.seed(123) before stochastic procedures to ensure reproducibility.

## Results

The study encompassed 57 central ICUs in secondary and higher-level comprehensive public hospitals across 16 cities in Anhui Province, China. Between May 6 and June 6, 2022, the parent prospective study enrolled 2,255 ICU patients from 57 hospitals in Anhui Province, China. Among these patients, 364 met the Sepsis-3 criteria and were screened for inclusion in the present secondary analysis. After excluding 5 patients who withdrew during follow-up and 11 patients with missing clinical or follow-up information, 348 patients were included in the final analytical cohort (Supplementary Figure S1). Among the sepsis patient cohort, cTnI was identified as the most deficient variable, with a deficiency rate of 26.7% (Supplementary Table S1).

### Derivation of clinical sepsis phenotypes

Based on k-means clustering, the three-class model including phenotypic cluster-derived Cluster A, Cluster B and Cluster C may be the best fit. The heat map matrix (Supplementary Figure S2), elbow method (Supplementary Figure S3), and cumulative distribution function curves (Supplementary Figure S4) indicate that the three-class model is optimal, and the tracking plots show that there is no significant separation between the K = 2, K = 3, and K = 4 clusters, which are more stable (Supplementary Table S2 and Supplementary Figure S5). Whereas, the clustering consensus plot shows that Clusters with higher cluster consensus (>0.8) for all clusters are preferred, so two, three or four clusters are optimal (Supplementary Figure S6). Three clusters typing was visualized for PCA plots, the Silhouette Coefficient for K = 3 is 0.37 (Supplementary Figure S7), indicating moderate separation between clusters with some overlap among patients. Among the hierarchical clustering methods, the elbow method showed that the three-cluster model was optimal (Supplementary Figure S8), and the gap statistics method showed that the three-cluster model was optimal (Supplementary Figure S9). Cluster dendrograms were drawn using the split hierarchical clustering method (Supplementary Figure S10).

### Clinical phenotypes of sepsis based on K-means clustering

In our study, 348 patients with severe sepsis in Anhui Province were analyzed in an inferential cohort using K-means clustering, and the basic characteristics of the patients are summarized in Table 1. The median age of the patients was 73 years old, and there was a higher proportion of male patients, with males accounting for 60.5% of the total. Nearly half of the sepsis patients (48.9%) had a source of infection from the respiratory system, with respiratory infections topping the list of all sites of infection. The majority of sepsis patients, 46%, were from the emergency department.

**Table 1:** Characteristics of patients in the sepsis clinical phenotype derivation cohort.

Variables	Sepsis patients (*n* = 348)	Cluster A (*n* = 95)	Cluster B (*n* = 149)	Cluster C (*n* = 104)	*P*
Age (year)	73 (59, 80)	71 (55, 80)	71 (57, 80)	74 (65, 82)	0.021
Gender (male)	215 (60.5%)	62 (65.3%)	87 (58.4%)	66 (63.5%)	0.512
BMI (kg/m^2^)	22.5 (20.2, 24.7)	22.9 (20.4, 24.7)	22.5 (20.0, 24.8)	22.5 (20.5, 24.5)	0.654
Patients source					0.694
Emergency department	160 (46%)	46 (48.4%)	65 (43.6%)	49 (47.1%)	
Surgical ward	95 (27.3%)	24 (25.3%)	45 (30.2%)	26 (25.0%)	
Medical ward	82 (23.6%)	24 (25.3%)	34 (22.8%)	24 (23.1%)	
Other ICU	11 (3.2%)	1 (1.1%)	5 (3.4%)	5 (4.8%)	
Infection sites					0.033
Respiratory tract	170 (48.9%)	38 (40.0%)	77 (51.7%)	55 (52.9%)	
Abdominal	96 (27.6%)	36 (37.9%)	31 (20.8%)	29 (27.9%)	
Urinary	43 (12.4%)	19 (20.0%)	11 (7.4%)	13 (12.5%)	
Blood steam	29 (8.3%)	13 (13.7%)	9 (6.0%)	7 (6.7%)	
Intestine	27 (7.8%)	11 (11.6%)	8 (5.4%)	8 (7.7%)	
Bile duct	24 (6.9%)	10 (10.5%)	6 (4.0%)	8 (7.7%)	
Vital signs					
Body temperature (°C)	38.4 (37.5, 38.9)	38.5 (38.2, 39.0)	38.8 (38.4, 39.3)	37.0 (36.6, 37.6)	<0.001
Heart rate (bpm)	124.0 (104.0, 140.0)	135.0 (123.0, 144.0)	130.0 (123.0, 142.0)	89.0 (78.0, 100.8)	<0.001
Respiratory rate (bpm)	30.0 (21.0, 34.00)	34.0 (31.0, 36.0)	32.0 (29.8, 35.0)	18.0 (15.0, 21.0)	<0.001
Systolic pressure (mmHg)	112.0 (88.0, 138.0)	86.0 (77.0, 90.0)	142.0 (133.8, 152.0)	112.0 (102.0, 127.8)	<0.001
Laboratory tests					
WBC (×10^9^/L)	11.8 (7.2, 17.5)	11.9 (7.2, 19.8)	13.4 (9.1, 19.4)	9.9 (7.3, 14.3)	0.001
RBC (×10^12^/L)	3.5 (2.9, 4.2)	3.6 (2.9, 4.3)	3.5 (2.9, 4.3)	3.4 (2.9, 4.2)	0.779
Lymphocyte (×10^9^/L)	5.6 (3.4, 6.2)	3.7 (1.7, 8.6)	6.9 (3.1, 10.5)	4.5 (3.3, 7.4)	0.028
PLT (×10^9^/L)	137.0 (70.0, 216.0)	110.0 (62.0, 209.0)	178.0 (132.0, 274.8)	178.5 (119.3, 246.8)	<0.001
Na (mmol/L)	138.7 (134.6, 142.2)	138.2 (133.8, 142.7)	138.5 (133.7, 142.2)	139.0 (135.2, 142.0)	0.824
Cl (mmol/L)	103.0 (98.0, 107.8)	102.0 (96.1, 108.0)	103.7 (99.8, 108.2)	103.3 (100.1, 107.8)	0.236
BUN (mmol/L)	12.0 (8.0, 19.0)	15.5 (10.8, 19.0)	11.0 (7.7, 15.9)	14.1 (8.0, 22.4)	0.001
Cr (umol/L)	118.8 (71.1, 206.5)	193.0 (137.1, 271.2)	82.0 (63.9, 116.9)	103.9 (70.2, 165.8)	<0.001
CRP (mg/L)	116.6 (62.3, 188.6)	177.6 (112.3, 219.0)	179.8 (125.2, 221.0)	44.5 (13.8, 85.8)	<0.001
PCT (ng/mL)	6.4 (1.1, 25.1)	7.4 (1.7, 46.9)	3.2 (0.8, 23.0)	4.5 (0.7, 20.8)	0.023
ALB (g/L)	29.0 (24.8, 32.1)	28.4 (23.9, 32.0)	29.2 (25.3, 32.0)	29.8 (26.4, 33.2)	0.095
TBIL (umol/L)	18.0 (11.4, 32.8)	35.3 (22.3, 60.3)	13.3 (9.5, 18.3)	15.0 (10.7, 22.0)	<0.001
ALT (u/L)	24.0 (16.0,54.2)	34.0 (17.7, 100.0)	23.0 (15.5, 49.3)	22.4 (15.0, 46.5)	0.019
AST (u/L)	39.5 (23.7, 82.0)	65.0 (32.0, 150.0)	34.0 (24.7, 65.3)	39.0 (21.0, 60.5)	<0.001
ALP (u/L)	84.0 (62.0, 121.0)	84.0 (57.0, 123.0)	83.0 (62.0, 121.3)	84.0 (60.3, 140.0)	0.731
*γ*-GT (u/L)	36.0 (17.0, 73.3)	42.0 (15.0, 86.5)	35.0 (16.2, 86.3)	36.0 (23.3, 85.8)	0.395
PT (s)	14.7 (13.1, 17.0)	15.3 (13.3, 17.9)	14.5 (13.1, 16.4)	14.5 (12.8, 17.0)	0.154
APTT (s)	35.8 (30.0, 43.7)	38.0 (30.2, 44.7)	35.6 (29.4, 42.7)	36.1 (30.0, 42.8)	0.430
FIB (g/L)	4.2 (2.8, 5.6)	2.8 (1.9, 4.2)	5.8 (4.7, 7.3)	3.6 (2.8, 4.6)	<0.001
D-dimmer (ug/mL)	5.4 (2.5, 14.9)	5.8 (3.2, 16.5)	4.4 (2.3, 15.5)	5.2 (1.9, 13.6)	0.142
cTnI (ng/mL)	0.16 (0.03, 1.31)	0.19 (0.04, 1.35)	0.07 (0.02, 0.76)	0.18 (0.02, 0.77)	0.309
PH	7.4 (7.3, 7.4)	7.3 (7.2, 7.4)	7.4 (7.3, 7.5)	7.4 (7.3, 7.4)	0.012
PCO_2_ (mmHg)	35.0 (29.0, 42.0)	33.1 (28.0, 39.2)	34.0 (29.5, 39.2)	35.0 (29.8, 43.0)	0.397
Lac (mmol/L)	4.2 (2.5, 8.2)	5.0 (3.3, 11.9)	3.2 (2.0, 4.7)	3.4 (1.9, 6.5)	<0.001
PO_2_/FiO_2_ (mmHg)	244.0 (174.0, 303.0)	166.0 (135.0, 200.0)	304.8 (276.2, 373.1)	198.0 (168.8, 239.8)	<0.001
24 h urine output (mL)	1,783.3 (957.1, 2,333.3)	1,250.0 (953.3, 1,866.7)	1,333.3 (996.7, 2,103.3)	1,358.3 (996.7, 2,100.0)	0.221
Severity score					
APACHE II	21 (14, 26)	24.0 (19.0, 31.0)	16.0 (12.0, 24.0)	20.0 (15.0, 25.0)	<0.001
SOFA	9 (6, 12)	11.0 (7.0, 15.0)	7.0 (5.0, 10.0)	9.5 (6.0, 12.0)	<0.001
Charlson	4 (2, 8)	4.0 (3.0, 7.0)	3.0 (2.0, 6.0)	4.0 (2.0, 7.0)	0.028
Therapy					
Vasopressor	247 (71.0%)	92 (96.8%)	68 (45.6%)	87 (83.7%)	<0.001
Fluid resuscitation	265 (76.1%)	87 (91.6%)	90 (60.4%)	88 (84.6%)	<0.001
Mechanical ventilation	245 (70.4%)	79 (83.2%)	91 (61.1%)	75 (72.1%)	0.001
Outcomes					
Length of ICU stay	14 (7, 26)	18 (10, 28)	8 (5, 23)	11 (6, 25)	<0.001
28-day mortality	144 (41.4%)	64 (67.4%)	34 (22.8%)	46 (44.2%)	<0.001

BMI, body mass index; ICU, intensive care unit; WBC, white blood cell; RBC, red blood cell; PLT, platelet; Na, natrium; Cl, chloride; BUN, blood urea nitrogen; Cr, creatinine; CRP, C-reactive protein; PCT, procalcitonin; ALB, albumin; TBIL, total bilirubin; ALT, alanine aminotransferase; AST, aspartate aminotransferase; ALP, alkaline phosphatase; γ-GT, γ-glutamyl transpeptidase; PT, prothrombin time; APTT, activated partial thromboplastin time; FIB, fibrinogen; cTnI, cardiac troponin I; Lac, lactate; PO_2_/FiO_2_, arterial oxygen tension/fraction of inspiration O_2._

Based on K-means clustering, the cohort phenotype was deduced to be divided into three clusters with the optimal clustering model, which was divided into three clusters of Cluster A, Cluster B, and Cluster C in Table 1. Patients in Cluster A were characterized by a high inflammatory state, high metabolism, multiple organ impairment and inadequate tissue perfusion, while those in Cluster B were characterized by a high inflammatory state, high metabolism, compensated hepatic and renal function, and no significant lung injury. Patients in Cluster C were characterized by a low inflammatory state, low metabolism, hepatic and renal impairment, and mild lung injury. Accordingly, Cluster A was identified as a hyperinflammatory phenotype with severe systemic involvement and multiorgan dysfunction, Cluster B as a hyperinflammatory phenotype with relatively preserved hepatic and renal function, and Cluster C as a hypoinflammatory phenotype predominantly characterized by hepatorenal dysfunction. This study showed that Cluster A patients had the highest APACHE II, SOFA, and Charlson scores, and 71.0% of all sepsis patients always used vasoactive medications, with the highest use in Cluster A, where 96.8% of patients used vasoactive medications, 76.1% of sepsis patients received fluid resuscitation therapy, with Cluster A 91.6% of sepsis patients were treated with fluid resuscitation, 70.4% of sepsis patients were treated with mechanical ventilation, with the highest percentage in Cluster A sepsis patients, with 83.2% of sepsis patients mechanically ventilated, the 28-day mortality rate of sepsis patients was 41.4%, with a 28-day mortality rate of 67.4% in Cluster A, a 28-day mortality rate of 22.8% in Cluster B, and a 28-day mortality rate of 44.2% in Cluster C category.

### Evaluation of the efficacy of fluid resuscitation in a clinical phenotype cohort of sepsis based on K-means clustering

#### Primary outcome

Fluid resuscitation for sepsis patients in Cluster A 87 (91.6%), fluid resuscitation for sepsis patients in Cluster B 90 (60.4%), and fluid resuscitation for sepsis patients in Cluster C 88 (84.6%). After adjusting for potential confounders such as age, gender, Charlson Comorbidity Index (CCI), and Sequential Organ Failure Assessment (SOFA) scores, the clinical prognosis of the Cluster A group with fluid resuscitation was worse than the Cluster A group without fluid resuscitation Unadjusted RD for 28-day mortality 3.5% (95% CI, 1.2 to 5.8%), *P* = 0.003; adjusted RD for 28-day mortality 16.2% (95% CI, 1.4 to 31.0%), *P* = 0.032. Clinical outcomes associated with fluid resuscitation treatment in addition to the Cluster A group were not associated with the clinical outcomes of the other groups (Table 2). In Cluster A, fluid-resuscitation exposure was associated with higher 28-day mortality (adjusted RD, 16.2%; 95% CI, 1.4%–31.0%); however, the marked group imbalance (87 vs. 8 patients) warrants cautious, exploratory interpretation. For 28-day mortality, the interaction between fluid resuscitation and K-means-derived phenotype was statistically significant before adjustment (*P* for interaction = 0.014) and remained significant after adjustment for age, sex, Charlson Comorbidity Index, and SOFA score (*P* for interaction = 0.037) (Table 2). These findings suggested that the association between fluid resuscitation and 28-day mortality differed across the derived phenotypes.

**Table 2:** The association of fluid resuscitation on the primary outcome in each cluster.

Primary Outcome	Cluster A	*P*	Cluster B	*P*	Cluster C	*P*	*P* for interaction
28-day mortality (unadjusted risk difference)	3.5 (1.2, 5.8)	0.003	−6.9 (−41.1, 27.3)	0.692	−1.4 (−3.8, 1.0)	0.249	0.014
28-day mortality (adjusted risk difference^a^)	16.2 (1.4, 31.0)	0.032	−4.8 (−12.7, 3.1)	0.235	5.6 (−7.5, 18.7)	0.402	0.037

a Adjusting for age, sex, Charlson Comorbidity Index and Sequential Organ Failure Assessment (SOFA) scores.

#### Secondary outcomes

Mechanical ventilation was more common in septic patients in Cluster A receiving fluid resuscitation, with an unadjusted 28-day mortality RD of 5.2% (*P* = 0.011) and an adjusted RD of 6.7% (*P* = 0.003). Conversely, vasoactive drug use was less frequent in septic patients in Cluster B with fluid resuscitation, showing an unadjusted 28-day mortality RD of −3.6% (*P* = 0.003) and an adjusted RD of −5.1% (*P* = 0.025) (Table 3).

**Table 3:** The association of fluid resuscitation on the secondary outcomes in each cluster.

Secondary outcomes	Cluster A	*P*	Cluster B	*P*	Cluster C	*P*
Unadjusted risk difference						
Vasopressor	0.1 (−0.5, 0.6)	0.840	−3.6 (−6.0,−1.2)	0.003	1.3 (−3.2, 5.8)	0.570
Mechanical ventilation	5.2 (1.2, 9.2)	0.011	0.2 (−1.2, 1.6)	0.810	−1.3 (−7.5, 4.9)	0.680
Length of ICU stay	2.9 (−1.6, 7.4)	0.203	−0.1 (−0.3, 0.1)	0.210	−0.1 (−0.4, 0.2)	0.510
Adjusted risk difference						
Vasopressor	0.03 (−0.2, 0.2)	0.760	−5.1 (−9.6, −0.6)	0.025	0.1 (−0.2, 0.4)	0.380
Mechanical ventilation	6.7 (2.3, 11.1)	0.003	0.5 (−4.4, 5.5)	0.830	−0.3 (−1.1,0.5)	0.440
Length of ICU stay	1.6 (−7.3, 10.4)	0.730	−0.04 (−0.3, 0.2)	0.760	−4.5 (−12.7, 3.7)	0.280

#### Sensitivity analysis

Generalized estimating equations were used, adjusting for APACHE II and site of infection. Septic patients in Cluster A with fluid resuscitation had a worse prognosis. Specifically, their 28-day mortality unadjusted RD was 3.5% (95% CI, 1.2 to 5.8%), *P* = 0.003, and adjusted RD was 6.3% (95% CI, 0.3 to 12.3%), *P* = 0.040, showing a statistically significant difference (Table 4).

**Table 4:** The association of fluid resuscitation on the primary outcome in sensitivity analysis (adjusting for APACHE II and site of infection).

Primary outcome	Cluster A	*P*	Cluster B	*P*	Cluster C	*P*
28-day mortality (unadjusted risk difference)	3.5 (1.2, 5.8)	0.003	−6.9 (−41.1 27.3)	0.692	−1.4 (−3.8, 1.0)	0.249
28-day mortality (adjusted risk difference^a^)	6.3 (0.3, 12.3)	0.040	−3.6 (−11.1, 3.9)	0.351	3.1 (−8.4, 14.6)	0.597

a Adjusting for APACHE II and site of infection.

In the overlap weighting sensitivity analysis, adjusting for age, sex, Charlson Comorbidity Index, and SOFA score,baseline covariate balance between fluid resuscitation and non-fluid resuscitation groups was substantially improved after weighting (Supplementary Figure S11). The association between fluid resuscitation and higher 28-day mortality remained in Cluster A (adjusted RD 7.4%, 95% CI 0.4–14.4%, *P* = 0.038), while no significant associations were observed in Cluster B or Cluster C (Table 5).

**Table 5:** The association of fluid resuscitation on the primary outcome in sensitivity analysis (overlap weighting analysis).

Primary outcome	Cluster A	*P*	Cluster B	*P*	Cluster C	*P*
28-day mortality (unadjusted risk difference)	3.5 (1.2, 5.8)	0.003	−6.9 (−41.1 27.3)	0.692	−1.4 (−3.8, 1.0)	0.249
28-day mortality (adjusted risk difference^a^)	7.4 (0.4, 14.4)	0.038	−2.1 (−8.4, 4.2)	0.512	−1.6 (−8.2, 5.0)	0.634

a Adjusting for age, sex, Charlson Comorbidity Index and Sequential Organ Failure Assessment (SOFA) scores.

## Survival analysis of a sepsis clinical phenotype cohort based on K-means clustering

Kaplan–Meier survival analysis of the three clusters of sepsis patients, Cluster A, Cluster B and Cluster C. The 28-day mortality rate of sepsis patients Cluster A > Cluster C, Log-rank test *P* = 0.029, the difference was statistically significant in Figure 1A. In the cumulative hazard curve, the 28-day cumulative mortality rate of sepsis patients was higher in Cluster A group in Figure 1B.

**Figure 1:**
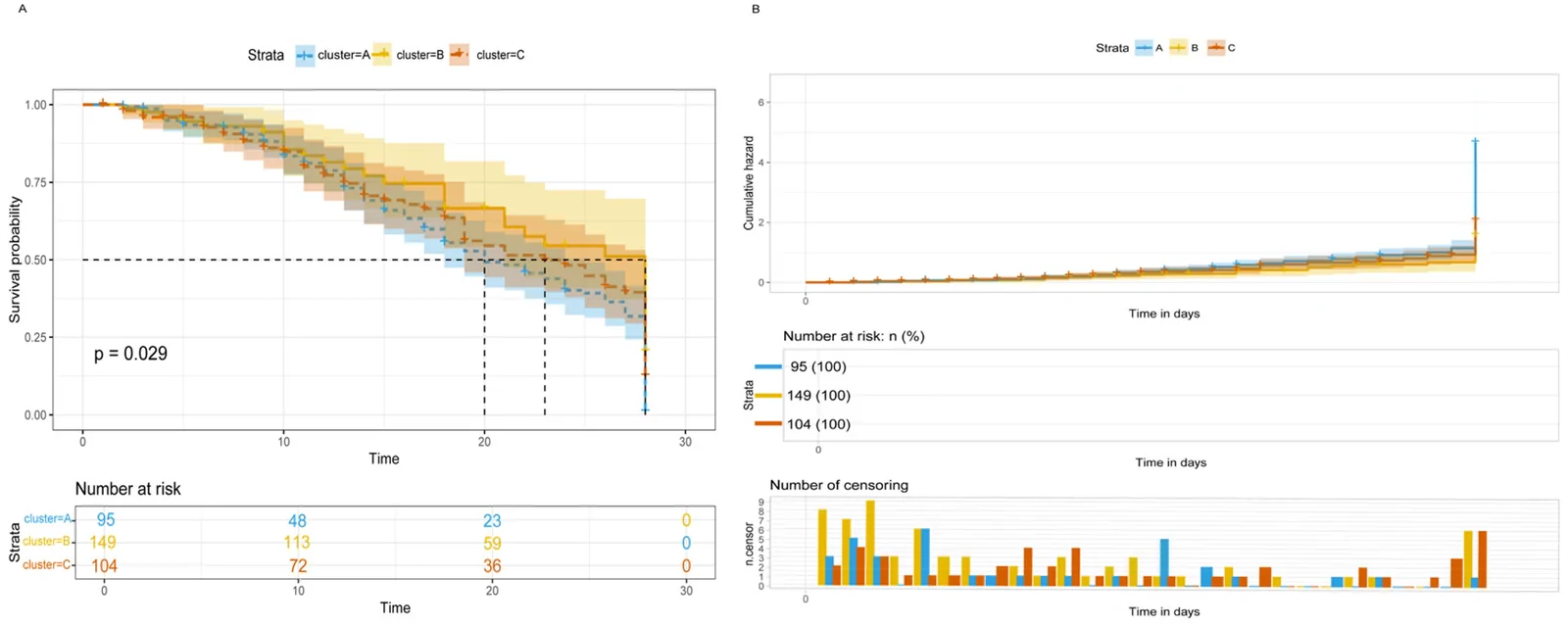
Survival analysis. **(A)** Survival analysis of Cluster A, Cluster B and Cluster C groups; **(B)** cumulative hazard of Cluster A, Cluster B and Cluster C groups.

## Clinical phenotypes of sepsis by hierarchical clustering methods

Based on the the hierarchical clustering methods, the optimal number of clusters was identified by the elbow method and the Gap statistic, and the cohort phenotype was deduced to be classified into three optimal clustering models, which were classified into the three clusters of Cluster 1, Cluster 2, and Cluster 3 (Supplementary Table S3). The three sets of clusters of Cluster 1, Cluster 2, and Cluster 3 and the K-means-based clustering method clustering characteristics and clinical outcomes were similar((Supplementary Table S3, S4, and S5). In the hierarchical clustering sensitivity analysis, the corresponding interaction *P* values were 0.001 before adjustment and 0.013 after adjustment (Supplementary Table S4). These findings suggested that the association between fluid resuscitation and 28-day mortality differed across the derived phenotypes. The survival analysis results were similar to K-means (Supplementary Figure S12).

## Discussion

The high heterogeneity of sepsis is a key factor limiting the consistency of treatment outcomes. Based on a multicenter prospective cohort in Anhui Province, this study employed unsupervised machine learning clustering methods to analyze routine clinical data available within 24 h of ICU admission. This approach identified three distinct clinical phenotypes of sepsis, each characterized by different clinical features and varying prognoses. Although the three-cluster solution yielded clinically interpretable phenotype patterns, the silhouette coefficient of 0.37 and the absence of clear separation among K = 2, K = 3, and K = 4 indicate considerable overlap between clusters. Therefore, these patterns should not be regarded as mutually exclusive or biologically validated sepsis subtypes.

Previous studies have revealed significant clinical heterogeneity in sepsis through phenotypic analysis. A retrospective ICU study categorized sepsis patients into four subtypes based on primary organ dysfunction: multiple organ dysfunction, neurological dysfunction, respiratory dysfunction, and mixed (11). The multiple organ dysfunction subtype exhibited the highest mortality rate. Another large multicenter retrospective study categorized sepsis patients into four α–δ phenotypes, with the δ phenotype—characterized by severe organ dysfunction and septic shock—carrying the highest short- and long-term mortality risk (6). Additionally, Bhavani et al. identified four subphenotypes based on early vital signs, some of which exhibited markedly elevated mortality risk potentially linked to increased vasopressor requirements (12). Although these studies provide important insights into sepsis heterogeneity and personalized management, most are retrospective or secondary analyses, inevitably subject to selection bias and limitations in data completeness.

Current research on clinical phenotypes of sepsis primarily originates from developed countries (13–16), with limited prospective multicenter evidence from low- and middle-income regions. Based on this, this study utilized a multicenter prospective observational cohort in Anhui Province and applied machine learning clustering methods to analyze routine clinical indicators available within 24 h of ICU admission. This identified three distinct sepsis clinical phenotypes characterized by different inflammatory states, metabolic features, and patterns of organ dysfunction. The three phenotype patterns identified in this study were: (1) Cluster A, characterized by high inflammation and metabolic activity, inadequate tissue perfusion, pulmonary injury, and multiorgan dysfunction; (2) Cluster B, characterized by high inflammation and metabolic activity, relatively preserved hepatic and renal function, and minimal pulmonary injury; and (3) Cluster C, characterized by relatively low inflammation and metabolic activity, predominant hepatic and renal dysfunction, and mild pulmonary injury. These patterns should be interpreted as overlapping, data-derived clinical patterns rather than discrete biological subtypes. These findings broadly align with previously reported “hyperinflammatory” and “hypovolemic” sepsis phenotypes (17, 18), further supporting the existence of clinical subtypes of sepsis with distinct immunological and pathophysiological characteristics.

Our study derives new clinical phenotypes based on machine learning clustering analysis. We observed that fluid resuscitation differed in clinical outcomes in different clustering groups, both in the K-means clustering method and the hierarchical clustering method, and a higher 28-day mortality rate could be observed in septic patients with fluid resuscitation than in those without fluid resuscitation in the Cluster A or Cluster 1 group, after unadjusted and adjusted for the risk differentials, instead of an increase in the frequency of the use of mechanical ventilation. A decrease in the use of vasoactive drugs in septic patients with fluid resuscitation compared to those without fluid resuscitation can be observed in the Cluster B or Cluster 3 groups. Some studies have been reported to show that fluid resuscitation therapy may not be beneficial, but rather increases the incidence of pulmonary edema and to some extent increases the use of mechanical ventilation (19–21). In a previous multicenter study, machine learning methods such as cluster analysis were used to classify clinical phenotypes into four categories; in the *δ* phenotype, liquid resuscitation therapy was harmful in more than half of the trials, and in the *α* phenotype, it was beneficial (6). *α* phenotypes in which liquid resuscitation therapy was beneficial were similar to those originally reported by Rivers et al (22). Our findings suggest that machine learning-derived clinical phenotypes may identify patient groups with different associations between fluid resuscitation and outcomes. And that it is difficult to distinguish what kind of sepsis liquid resuscitation is effective for using traditional methods. Machine learning unsupervised derivation of new clinical phenotypes has an important role in heterogeneous treatment of sepsis. However, Fluid resuscitation was clinician-directed and generally prompted by hypotension, elevated lactate, or suspected hypoperfusion; therefore, patients receiving fluids may have had greater unmeasured hemodynamic severity. Conversely, non-resuscitated patients may have lacked a hypoperfusion indication or had concerns regarding fluid intolerance, pre-existing volume overload or heart failure, limitations of care, or deterioration too rapid for intervention. Because these treatment-selection factors were not systematically recorded, adjustment for SOFA, CCI, APACHE II, and overlap weighting cannot fully address confounding by indication or reverse causation; therefore, the findings should be interpreted as associations rather than causal effects.

Our study sepsis patients were more critical with a median APACHE II score of 21 and a median SOFA of 9. It has been reported that sepsis patients in developed countries are less critical than in our study (23). There are studies in which the criticality of septic patients in developed countries is similar to our study, but the mortality rate in our study is much higher than the reported centers (24). Many influencing factors such as time from onset to consultation, level of awareness of the disease, economic level and criticality of the disease may have contributed to the low mortality rate in developed countries, which was much higher in our study (25, 26).

This study has several limitations. First, despite employing the random forest method to handle missing data, potential bias could not be entirely avoided. Second, due to limited central monitoring conditions, immunological and molecular biological indicators could not be systematically incorporated. Third, although the associations varied across phenotypes, the observational design precludes causal inference. Because fluid resuscitation was clinician-directed, which may have introduced confounding by indication and reverse causation. Residual confounding by indication and reverse causation cannot be excluded despite statistical adjustment. Meanwhile, Cluster A was markedly imbalanced (87 exposed vs. 8 unexposed), resulting in limited treatment overlap and an imprecise risk-difference estimate. Therefore, despite consistent sensitivity analyses, this finding should be considered exploratory rather than confirmatory. Fourth, although cluster stability was assessed using consensus clustering and hierarchical clustering, patient-level bootstrap validation was not performed. Further external validation is needed to confirm the reproducibility and generalizability of the identified phenotypes. Finally, multiple comparisons were performed without formal adjustment, so the reported *P* values should be interpreted as nominal and exploratory. We have acknowledged the potential inflation of type I error due to multiple comparisons as an important limitation of this study.

## Conclusions

In this multicenter study, machine learning–based clustering identified three distinct clinical phenotypes of sepsis with different prognoses and heterogeneous responses to fluid resuscitation. However, these exploratory observational findings do not establish phenotype-specific causal effects or justify changes in fluid-resuscitation practice, and further prospective studies are required to validate their clinical utility.

## Data Availability

The raw data supporting the conclusions of this article will be made available by the authors, without undue reservation.
