# Supplementary material for: Derivation of a novel clinical phenotype of sepsis in critically ill patients: a secondary analysis of a multicentre, prospective, observational study
Source: Front Med (Lausanne). 2026 Sep 16;13:1910023. doi: 10.3389/fmed.2026.1910023 (PMC13624142; doi:10.3389/fmed.2026.1910023)
Supplement: Supplementary file 1 [file Supplementaryfile2.docx]

**Supplemental Figure legends**

**Supplemental Figure 1. Flowchart of this study**

Between May 6 and June 6, 2022, a total of 2,255 ICU patients were enrolled from 57 hospitals in Anhui Province, China. Among them, 364 patients met the Sepsis-3 diagnostic criteria and were screened for inclusion in this secondary analysis. Sixteen patients were excluded, including 5 patients who withdrew from the study and 11 patients with important missing clinical or follow-up information. The remaining 348 patients constituted the final analytical cohort. These patients were included in the primary K-means clustering analysis and the supplementary hierarchical clustering analysis for phenotype derivation and methodological comparison.

**Supplemental Figure 2. Consensus matrix heatmaps based on number of clusters**

Consensus clustering generates a consistency matrix for each K-value, where both rows and columns represent samples, and each value in the matrix indicates how often a pair of samples is clustered together over multiple resamplings and clustering (from 0 to 1), where 0 means that two data items are never clustered together and 1 means that two data items are always clustered together, with the magnitude of the frequency indicated by the color shade. The consistency matrix is sorted according to the results of the clustering, which are represented as a tree on top of the heat map, with different categories marked using different colors. The first figure in the upper left corner is the legend of the heatmap, the next five figures show the heatmap of the consistency matrix for different values of K (number of categories). Based on the examination of the consensus clustering diagrams, the “optimal” number of clusters is chosen to be K=2 or 3.

**Supplemental Figure 3. Elbow method**

The elbow method shows the variance ratio (%) as a function of the number of clusters. The delta-area plot shows the relative change in area under the CDF curve for K and K-1 compared to K. Larger values indicate the superiority of the clustering effect for K values compared to K-1 values. If the change in the area of neighboring K values is small, it indicates that further increasing the K value will not significantly improve the clustering stability, and it can be seen that the change in the area of neighboring K values is small when K=3.

**Supplemental Figure 4. Consensus CDF**

The Consensus Cumulative Distribution Function (Consensus CDF) is used to assess the stability of the clustering results for different number of clusters K. The CDF is used to assess the stability of the clustering results for different number of clusters. It reflects the stability and validity of clustering by showing the cumulative distribution of sample consistency under different values of K. Generally speaking, when the CDF distribution is smoother and its value is higher, it indicates that the clustering results are more robust and the cluster consistency of the samples is stronger under this value of K. The enhancement of the area under the CDF curve can also be used as an important indicator to assess the effectiveness of the clustering - the larger the area under the curve, the better the stability and consistency of the clustering results. The figure demonstrates the CDF distribution under different number of clusters K. When K ≥ 3, the CDF curve becomes flatter, indicating that the stability of the clustering results is better at this point and the consistency of clustering between samples is higher. However, when K=4, the enhancement effect of the area under the CDF curve is relatively small, suggesting that further increasing the value of K has a limited effect on the improvement of the clustering results. According to the results of CDF analysis, K=3 is considered the optimal clustering number.

**Supplemental Figure 5.Tracking plot**

The tracking plot displays changes in patient cluster assignments across candidate values of K. Assignments changed across K=2, K=3, and K=4, and the plot did not show unequivocal separation favoring a single solution. Thus, selection of K=3 was based on the combined clustering diagnostics and clinical interpretability rather than clear superiority in the tracking plot.

**Supplemental Figure 6. Cluster-consensus**

Item-consensus (IC) is a measure of the consistency between a sample and the cluster to which it belongs, and it indicates the average consistency value of a sample within a cluster. Specifically, a higher IC value indicates that the sample is more similar to other samples within the clustering cluster, indicating a more reasonable clustering result. Under different number of clusters K, each sample will have corresponding multiple IC values, and these IC values reflect the clustering effect of the sample under different number of clusters.Cluster-consensus (CLC) is the average of the consistency among all samples within a cluster.The higher the CLC value, the higher the similarity among the samples within the cluster, and the more stable the clustering result.The CLC value helps us to evaluate the degree of improvement of the clustering effect when the value of K is increased. By comparing the CLC values at different values of K, we are able to determine how much the clustering quality improves.In this study, by calculating the IC and CLC values under different K values, we find that the clustering effect performs optimally when K=2, K=3 or K=4. The specific numerical and graphical results are shown in Table 2 and Figure 5, and these results indicate that the stability and consistency of clustering reaches a high level when the number of clusters is 2, 3 or 4, which is suitable for further analysis.

Supplemental Figure 7. Principal-component visualization and silhouette assessment for K=3. The silhouette coefficient was 0.37, indicating only moderate separation and substantial overlap among patients; the three clusters should therefore be interpreted as overlapping data-derived phenotype patterns rather than sharply discrete classes.

**Supplemental Figure 8. Elbow method for identifying the optimal number of clusters through divisive hierarchical clustering**

The Elbow Method (EM) is a classical cluster analysis technique for determining the optimal number of clusters K. The basic idea is to plot K versus SSE by calculating the SSE (sum of squared errors, sum of squared errors), i.e., the sum of the squares of the distances of the data points from their respective cluster centers for different number of clusters, for different values of K. The SSE is calculated for each cluster by calculating the sum of squares of the distances of the data points from their respective cluster centers for different values of K and plotted against the SSE. As the value of K increases, the SSE decreases as more clusters fit the data better. However, as the value of K continues to increase, the decrease in SSE gradually slows down, eventually forming an inflection point, similar to the shape of an elbow. The K value corresponding to this “elbow” is usually considered the optimal number of clusters, and the number of clusters corresponding to the elbow is the optimal number of clusters.

In this study, split hierarchical clustering is used to calculate SSE by using the total.withinss parameter in its output, and the K value is plotted as a function of SSE. The graph shows that when K=3, the decreasing trend of SSE starts to become flat and close to a straight line, which indicates that K=3 is a reasonable choice. At this K value, a good balance between tightness and separation of data clustering is achieved. Therefore, the optimal number of clusters was determined to be 3 best based on the elbow method.

**Supplemental Figure 9. Gap statistic method**

In this study, the quality of clustering was assessed by calculating the Gap statistic at different values of K. The Gap statistic is a metric used to assess the effectiveness of clustering by randomizing the original dataset many times, generating multiple zero-reference datasets, and calculating the difference in the distances between the original dataset and the randomized dataset.A larger Gap statistic indicates a better clustering result. The computed results of Gap statistic under different values of K are plotted as a function of K value. By comparing the Gap statistic at different values of K, the optimal number of clusters can be determined. The graph shows that the Gap statistic reaches its maximum value at K=3, indicating that K=3 is the optimal number of clusters. To further validate the result, the Gap statistic can be calculated using the cluster::clusGap function to ensure the accuracy of its calculation process. Ultimately, based on the analysis of the Gap statistic, the study determined the optimal number of clusters to be three. This result provides an important theoretical basis for the subsequent clustering analysis, showing that K=3 can effectively divide the different classes in the dataset.

**Supplemental Figure 10. Cluster dendrogram using divisive hierarchical clustering**

Divisive hierarchical clustering performs clustering using a top-down strategy. Starting with all data points as a whole cluster, the cluster is then gradually split into smaller clusters until each data point becomes an independent cluster or other stopping conditions are met. At each step, hierarchical clustering decides how to merge the clusters by calculating the distance between each sample or cluster and the other samples or clusters in the data set. Specifically, hierarchical clustering algorithms typically construct cluster trees (or dendrograms) using measures such as minimum distance, maximum distance, or average distance. This process is performed iteratively, and the resulting clustering tree clearly demonstrates the hierarchical relationships between data samples.

In this study, patients with sepsis were categorized into 3 main clusters through hierarchical clustering. These three clusters represent significant differences in certain clinical characteristics of sepsis patients, which helps to further analyze their different clinical manifestations and prognoses. The clustering dendrogram shows the results of hierarchical clustering of sepsis patients, which helps to visualize and understand the relationship between the clusters and the clustering process.

**Supplemental Figure 11. Covariate balance before and after overlap weighting**

Absolute standardized mean differences (SMDs) for age, sex, Charlson Comorbidity Index (CCI), and Sequential Organ Failure Assessment (SOFA) score between patients who received and did not receive fluid resuscitation are shown before and after overlap weighting. Blue points and lines represent the unadjusted estimates, whereas yellow points and lines represent the estimates after propensity score–based overlap weighting. The vertical dashed line indicates an absolute SMD of 0.10, which was used as the threshold for acceptable covariate balance. Overlap weighting substantially reduced the absolute SMDs for all four covariates, indicating improved baseline comparability between the fluid-resuscitation and non-fluid-resuscitation groups. The propensity score model included age, sex, CCI, and SOFA score.

**Supplemental Figure 12. Survival analysis of a sepsis clinical phenotype cohort based on divisive hierarchical clustering**

Survival analysis of Cluster 1, Cluster 2 and Cluster 3 groups; (b)Cumulative hazard of Cluster 1, Cluster 2 and Cluster 3 groups

This figure presents the 28-day survival outcomes of patients classified into three clinical phenotypes using divisive hierarchical clustering based on the selected clinical variables. The follow-up period began at ICU admission and continued for 28 days.

(A) Kaplan–Meier survival curves for Cluster 1, Cluster 2, and Cluster 3, showing the probability of remaining alive over time.
(B) Cumulative hazard curves for the three clusters, illustrating the cumulative risk of death during the 28-day follow-up period. Differences in survival distributions were assessed using the log-rank test. This analysis served as a methodological sensitivity analysis to compare the hierarchical clustering results with the phenotypes derived using K-means clustering.
