# Supplementary material for: Derivation of a novel clinical phenotype of sepsis in critically ill patients: a secondary analysis of a multicentre, prospective, observational study
Source: Front Med (Lausanne). 2026 Sep 16;13:1910023. doi: 10.3389/fmed.2026.1910023 (PMC13624142; doi:10.3389/fmed.2026.1910023)
Supplement: Supplementary file 2 [file Table1.docx]

| **Supplemental Table 1. Deriving missing values for datasets** | |
| --- | --- |
| **variables** | **Derivation cohort（n=348）** |
| HR | 2（0.6%） |
| 24 hour urine output | 88（25.3%） |
| Lymphocyte count | 42（12.1%） |
| PLT | 22（6.3%） |
| Na | 47（13.5%） |
| Cl | 56（16.1%） |
| BUN | 46（13.2%） |
| Cr | 46（13.2%） |
| CRP | 51（14.7%） |
| PCT | 70（20.1%） |
| ALB | 63（18.1%） |
| TBIL | 29（8.3%） |
| ALT | 30（8.6%） |
| AST | 39（11.2%） |
| ALP | 62（17.8%） |
| γ-GT | 62（17.8%） |
| PT | 37（10.6%） |
| APTT | 33（9.5%） |
| FIB | 34（9.8%） |
| D-Dimer | 48（13.8%） |
| cTnI | 93（26.7%） |
| PH | 38（10.9%） |
| Lac | 48（13.8%） |
| PO_2_/FiO_2_ | 76（21.8%） |
| HR: Heart rate, PLT: Platelet, Na: Natrium, Cl: Chlorine, BUN: Blood urea nitrogen, Cr: Creatinine, CRP: C-Reactive Protein, PCT: Procalcitonin, ALB: Albumin, TBIL: Total bilirubin, ALT: Alanine aminotransferase, AST: Aspartate aminotransferase, ALP: Alkaline phosphatase, γ-GT: Gamma-glutamyl transferase, PT: Prothrombin time, APTT: Activated partial thromboplastin time, FIB: Fibrinogen, cTnI: Cardiac troponin I, PCO_2_: Partial pressure of carbon dioxide, Lac: Lactate, PO_2_/FiO_2_: Arterial oxygen tension/ Fraction of inspiration O_2_. | |

| **Supplemental Table 2. Clustering consistency** | | |
| --- | --- | --- |
| **K** | **cluster** | **Cluster Consensus** |
| 2 | 1 | 0.796323529 |
| 2 | 2 | 0.904435941 |
| 3 | 1 | 0.838174603 |
| 3 | 2 | 0.787698413 |
| 3 | 3 | 0.825198413 |
| 4 | 1 | 1.000000000 |
| 4 | 2 | 0.787698413 |
| 4 | 3 | 0.761177249 |
| 4 | 4 | 0.739413265 |
| 5 | 1 | 0.520634921 |
| 5 | 2 | 0.726984127 |
| 5 | 3 | 0.861904762 |
| 5 | 4 | 0.629889456 |
| 5 | 5 | 1.000000000 |

| **Supplemental Table 3. Characteristics of the sepsis patients (Based on the the hierarchical clustering methods)** | | | | | |
| --- | --- | --- | --- | --- | --- |
| **variables** | **Sepsis patients**  **(n=348 )** | **Cluster 1**  **(n=141 )** | **Cluster 2**  **(n=129)** | **Cluster 3**  **(n=78 )** | **P** |
| **Age (year)** | 73 (59, 80) | 71 (55, 80) | 75 (67, 81) | 71 (60, 80) | 0.011 |
| **Sex (male)** | 215 (60.5%) | 82 (58.2%) | 85 (65.9%) | 48 (61.5%) | 0.429 |
| **BMI (kg/m^2^)** | 22.5 (20.2, 24.7) | 22.4 (21.2, 25.6) | 22.1 (20.2, 24.7) | 21.4 (20.1, 25.2) | 0.791 |
| **Patients source** |  |  |  |  | 0.536 |
| emergency department | 160 (46%) | 65 (43.6%) | 49 (47.1%) | 46 (48.4%) |  |
| surgical ward | 95 (27.3%) | 45 (30.2%) | 26 (25.0%) | 24 (25.3%) |  |
| medical ward | 82 (23.6%) | 34 (22.8%) | 24 (23.1%) | 24 (25.3%) |  |
| other ICU | 11 (3.2%) | 7 (5.0%) | 3 (2.3%) | 1 (1.3%) |  |
| **Infection sites** |  |  |  |  | 0.067 |
| respiratory tract | 170 (48.9%) | 71 (50.4%) | 63 (48.8%) | 36 (46.2%) |  |
| abdominal | 96 (27.6%) | 41 (29.1%) | 36 (27.9%) | 19 (24.5%) |  |
| urinary | 43 (12.4%) | 17 (12.1%) | 15 (11.6%) | 11 (14.1%) |  |
| blood steam | 29 (8.3%) | 11 (7.8%) | 8 (6.2%) | 10 (12.8%) |  |
| intestine | 27 (7.8%) | 12 (8.5%) | 8 (6.2%) | 7 (9.0%) |  |
| Bile duct | 24 (6.9%) | 11 (7.8%) | 7 (5.4%) | 6 (7.7%) |  |
| **Vital signs** |  | | | | |
| body temperature (℃) | 38.4(37.5,38.9) | 38.5 (38.1, 39.0) | 37.0 (36.7, 37.5) | 38.9 (38.4, 39.3) | ＜0.001 |
| Heart rate (bpm ) | 124.0(104.0, 140.0) | 133.0 (124.0, 146.0) | 89.0 (78.0, 100.8) | 136.0 (127.0, 146.0) | ＜0.001 |
| respiratory rate (bpm ) | 30.0 (21.0,34.00) | 33.0 (29.0, 36.0) | 18.0 (15.0, 21.0) | 32.0 (29.0, 35.0) | ＜0.001 |
| systolic pressure (mmHg ) | 112.0(88.0,138.0) | 90.0 (78.8, 101.3) | 114.0 (103.0, 128.) | 144.0 (136.0, 154.0) | ＜0.001 |
| **Laboratory tests** |  | | | | |
| WBC(×10^9^/L) | 11.8 (7.2,17.5) | 13.5 (9.8, 19.4) | 10.5 (7.5, 14.3) | 11.7 (7.1, 20.0) | ＜0.001 |
| RBC(×10^12^/L) | 3.5 (2.9,4.2) | 3.4 (3.0, 4.5) | 3.3 (2.8, 4.1) | 3.4 (2.9, 4.4) | 0.439 |
| Lymphocyte (×10^9^/L) | 5.6 (3.4,6.2) | 3.0 (2.1, 7.6) | 4.9 (3.0, 7.4) | 6.7 (3.9, 11.2) | 0.031 |
| PLT (×10^9^/L) | 137.0 (70.0,216.0) | 177.0 (120.0, 243.0) | 178.5 (119.3, 246.8) | 194.0 (146.0, 277.0) | ＜0.001 |
| Na (mmol/L) | 138.7 (134.6,142.2) | 138.5 (134.4, 147.5) | 139.0 (136.1, 145.0) | 137.2 (133.6, 145.5) | 0.469 |
| Cl (mmol/L) | 103.0 (98.0,107.8) | 104.5 (97.8, 110.3) | 101.0 (98.4, 109.2) | 102.3 (97.1, 107.8) | 0.736 |
| BUN (mmol/L) | 12.0 (8.0,19.0) | 14.8 (8.3, 22.8) | 10.7 (7.6, 15.3) | 15.4 (11.4.19.0.0) | ＜0.001 |
| Cr (umol/L) | 118.8 (71.1, 206.5) | 165.0 (92.9, 273.9) | 111.4 (70.8, 166.0) | 79.2 (64.0, 111.0) | ＜0.001 |
| CRP (mg/L) | 116.6 (62.3, 188.6) | 185.3 (126.0, 233.0) | 82.1 (51.4, 116.6) | 172.3 (108.0, 215.7) | ＜0.001 |
| PCT (ng/ml) | 6.4 (1.1,25.1) | 7.0 (1.4, 41.9) | 4.5 (0.7, 20.4) | 2.6 (0.7, 20.0) | 0.015 |
| ALB (g/L) | 29.0 (24.8,32.1) | 27.2 (24.5, 33.0) | 28.8 (25.1, 34.2) | 28.2 (26.9, 34.0) | 0.086 |
| TBIL (umol/L) | 18.0 (11.4,32.8) | 26.1 (15.6, 44.6) | 15.0 (10.7, 22.0) | 12.6 (9.4, 18.0) | ＜0.001 |
| ALT (u/L) | 24.0 (16.0,54.2) | 27.0 (20.0, 75.9) | 24.4 (18.0, 56.5) | 21.0 (16.2, 44.0) | 0.215 |
| AST (u/L) | 39.5 (23.7,82.0) | 68.0 (30.6, 231.5) | 35.0 (24.6, 70.5) | 30.4 (22.7, 40.0) | ＜0.001 |
| ALP (u/L) | 84.0 (62.0,121.0) | 103.0 (82.0, 284.3) | 81.0 (55.6, 150.0) | 54.0 (29.4, 76.0) | 0.041 |
| γ-GT (u/L) | 36.0 (17.0,73.3) | 47.0 (21.5, 95.3) | 35.0 (23.0, 82.7) | 29.8 (14.0, 56.0) | 0.052 |
| PT (s) | 14.7 (13.1,17.0) | 14.5 (13.1, 16.4) | 14.5 (12.8, 17.0) | 15.3 (13.3, 17.9) | 0.154 |
| APTT (s) | 35.8 (30.0,43.7) | 41.6 (39.0, 72.9) | 36.0 (30.6, 52.8) | 30.1 (25.3, 40.6) | 0.736 |
| FIB (g/L) | 4.2 (2.8,5.6) | 2.9 (2.0, 4.7) | 3.6 (2.9, 5.5) | 5.6 (4.4, 8.9) | ＜0.001 |
| D-dimmer (ug/ml) | 5.4 (2.5,14.9) | 5.4 (3.3, 17.5) | 5.2 (2.8, 12.4) | 5.7 (3.2, 16.5) | 0.568 |
| cTnI (ng/ml) | 0.16 (0.03, 1.31) | 0.20 (0.05, 1.63) | 0.22 (0.02, 0.85) | 0.01 (0.00, 0.45) | 0.009 |
| PH | 7.4 (7.3,7.4) | 7.4 (7.3, 7.5) | 7.4 (7.3, 7.4) | 7.3 (7.2, 7.4) | 0.012 |
| PCO_2_ (mmHg) | 35.0 (29.0,42.0) | 34.0 (30.6, 44.0) | 35.0 (28.6, 48.9) | 33.8 (30.0, 39.2) | 0.352 |
| Lac (mmol/L) | 4.2 (2.5,8.2) | 4.7 (2.6, 8.3) | 3.2 (1.6, 7.3) | 3.2 (1.95, 4.3) | ＜0.001 |
| PO_2_/FiO_2_ (mmHg) | 244.0(174.0,303.0) | 183.6 (143.8, 255.5) | 198.0 (168.8, 239.8) | 322．0 (289.0, 389.0) | ＜0.001 |
| 24 hour urine output (ml) | 1783.3(957.1,2333.3) | 1000.4 (896.7, 2754.8) | 1428.9 (1063.6, 2607.5) | 2250.0 (1153.3, 3484.3) | 0.318 |
| **Severity score** |  | | | | |
| APACHE II | 21 (14, 26) | 25.0 (19.0, 33.0) | 20.0 (16.0, 27.0) | 18.0 (14.0, 22.0) | ＜0.001 |
| SOFA | 9 (6, 12) | 11.0 (6.0, 17.0) | 10.0 (5.0.15.0) | 8.0 (6.0, 12.0) | ＜0.001 |
| Charlson | 4 (2, 8) | 4.0 (3.0, 9.0) | 4.0 (2.0, 7.0) | 3.0 (2.0, 6.0) | 0.006 |
| **Therapy** |  | | | | |
| vasopressor | 247 (71.0%) | 125 (88.7%) | 98 (76.0%) | 24 (30.8%) | ＜0.001 |
| fluid resuscitation | 265 (76.1%) | 131 (92.9%) | 84 (65.1%) | 50 (64.1%) | ＜0.001 |
| fluid resuscitation dosage(ml) | 1802.0(1500.0,2110.0) | 1980.5(1830.5,2100.5) | 1900.0(1750.5,2070.0) | 1750.5(1700.0,1980.0) | <0.001 |
| mechanical ventilation | 245 (70.4%) | 122 (86.5%) | 94 (72.9%) | 29 (37.2%) | ＜0.001 |
| **Outcomes** |  |  |  |  |  |
| length of ICU stay | 14 (7,26) | 16.5 (7,28) | 15 (6,20) | 8 (6,13) | ＜0.001 |
| 28-day mortality | 144 (41.4%) | 85 (60.3%) | 49 (38.0%) | 10 (12.8%) | ＜0.001 |
| BMI, body mass index; ICU, intensive care unit; WBC, white blood cell; RBC, Red blood cell; PLT, platelet; Na, Natrium; Cl, Chloride; BUN, Blood urea nitrogen; Cr, Creatinine; CRP, C-reactive protein; PCT, procalcitonin; ALB, Albumin; TBIL, Total bilirubin; ALT, Alanine aminotransferase ; AST, Aspartate aminotransferase ; ALP, alkaline phosphatase; γ-GT, γ-glutamyl transpeptidase; PT, prothrombin time; APTT, activated partial thromboplastin time; FIB, Fibrinogen; cTnI, Cardiac troponin I; Lac, Lactate; PO_2_/FiO_2_, Arterial oxygen tension/Fraction of inspiration O_2._ | | | | | |

|  | **Supplemental Table 4. The Association of Fluid Resuscitation on the Primary Outcome in Each Cluster (Based on the the hierarchical clustering methods).** | | | | | | | |
| --- | --- | --- | --- | --- | --- | --- | --- | --- |
| Primary Outcome | | Cluster 1 | P | Cluster 2 | P | Cluster 3 | P | P for interaction |
| 28-day mortality (unadjusted risk difference) | | 3.6 (1.5, 5.7) | ＜0.001 | -3.6 (-9.3, 2.1) | 0.218 | -1.5 (-3.3, 0.3) | 0.107 | 0.001 |
| 28-day mortality (adjusted risk difference*) | | 7.8 (0.2, 15.4) | 0.045 | -10.5 (-106.9, 85.9) | 0.831 | -6.7 (-24.0, 10.6) | 0.447 | 0.013 |
|  | *adjusting for age, sex, Charlson Comorbidity Index and Sequential Organ Failure Assessment (SOFA) scores | | | | | | | |

| **Supplemental Table 5. The Association of Fluid Resuscitation on the Secondary outcomes in Each Cluster (Based on the the hierarchical clustering methods).** | | | | | | |
| --- | --- | --- | --- | --- | --- | --- |
| Secondary outcomes | Cluster 1 | P | Cluster 2 | P | Cluster 3 | P |
| **unadjusted risk difference** |  |  |  |  |  |  |
| vasopressor | 1.3 (-0.7, 3.3) | 0.201 | 1.1 (-0.1, 2.3) | 0.081 | -2.6 (-4.4, -0.8) | 0.005 |
| mechanical ventilation | 2.9 (1.1, 4.7) | 0.002 | 0.2 (-0.2, 0.7) | 0.323 | -0.7 (-3.0, 1.6) | 0.554 |
| length of ICU stay | 4.3 (-0.8, 9.4) | 0.098 | 1.4 (-1.1, 3.9) | 0.263 | -0.7 (-3.6, 2.2) | 0.626 |
| **adjusted risk difference** |  |  |  |  |  |  |
| vasopressor | 0.6 (-0.89, 2.01) | 0.448 | 0.2 (-0.2, 0.6) | 0.305 | -1.2 (-2.3, -0.1) | 0.034 |
| mechanical ventilation | 1.6 (0.1, 3.1) | 0.041 | 0.8 (-15.6, 17.2) | 0.923 | -1.1 (-5.4, 3.3) | 0.632 |
| length of ICU stay | 3.7 (-9.8, 17.2) | 0.592 | -0.5 (-1.2, 0.2) | 0.142 | -4.5 (-22.4, 13.4) | 0.623 |

| **Supplemental Table 6. The Association of Fluid Resuscitation on the Primary Outcome in Sensitivity analysis (Based on the the hierarchical clustering methods).** | | | | | | |
| --- | --- | --- | --- | --- | --- | --- |
| Primary Outcome | Cluster 1 | P | Cluster 2 | P | Cluster 3 | P |
| 28-day mortality (unadjusted risk difference) | 4.7 (1.9, 7.5) | ＜0.001 | -1.4 (-6.7, 3.9) | 0.603 | -2.3 (-42.2, 37.6) | 0.910 |
| 28-day mortality (adjusted risk difference*) | 1.9 (0.5, 3.3) | 0.006 | -5.7 (-20.2, 8.8) | 0.442 | -0.09 (-0.25, 0.07) | 0.285 |
| *adjusting for APACHE II and site of infection | | | | | | |
